# Diversity and Relative Abundance of Ungulates and Other Medium and Large Mammals in Flooded Forests in the Dahomey Gap (Togo)

**DOI:** 10.3390/ani12213041

**Published:** 2022-11-04

**Authors:** Gabriel Hoinsoudé Segniagbeto, Kokouvi Gbétey Akpamou, Yawo Konko, John Kokou Toviho Gaglo, Guillaume Koffivi Ketoh, Daniele Dendi, Julia Elizabeth Fa, Luca Luiselli

**Affiliations:** 1 Laboratory of Ecology and Ecotoxicology, Faculty of Sciences, University of Lomé, Lomé 01BP1515, Togo; 2 Laboratory of Forestry Research, Faculty of Sciences, University of Lomé, Lomé 01BP1515, Togo; 3 Ministry of Agriculture, Livestock and Rural Development, Lomé BP305, Togo; 4 Togolese Society for Nature Conservation (AGBO-ZEGUE NGO), Lomé 06BP6057, Togo; 5 Institute for Development, Ecology, Conservation and Cooperation, via G. Tomasi di Lampedusa 33, I-00144 Rome, Italy; 6 Department of Animal and Environmental Biology, Rivers State University of Science and Technology, Port Harcourt 500101, Nigeria; 7 Department of Natural Sciences, School of Science and the Environment, Manchester Metropolitan University, Manchester M15 6BH, UK

**Keywords:** mammalia, ungulates, community structure

## Abstract

**Simple Summary:**

One of the most important vegetation zones in West Africa is the so-called “Dahomey Gap”, which is a mostly savannah region that separates two rainforest blocks, the West African one (scientifically known as the Upper Guinean Forest block) and the central African one (Lower Guinean Forest block). Forest patches are sparsely distributed within the Dahomey Gap region. In this paper, we studied medium and large mammals (especially ungulates) within an area dominated by patches of flooded forests near the Mono river in south-eastern Togo. We described the species richness of mammals in this area. We found that 19 species coexisted in these forests. The number of species in each forest area correlated with the size of the forest patch. We stress that to ensure the protection of the Dahomey Gap mammals, we should seriously consider protecting not only the forest patches but also the surroundings, mainly savannah landscapes.

**Abstract:**

“The Dahomey Gap” is a human-derived mostly savannah region that separates the Guineo-Congolian rainforest block into two major units: the Upper Guinean and the Lower Guinean Forest blocks. Several forest patches are distributed throughout this savannah-dominated habitat. The mammal communities in the Dahomey Gap region have been poorly studied. In this paper we analyse the species richness and abundance of, as well as conservation implications for, medium and large mammals (especially ungulates) inhabiting a complex of flooded forests near the Mono river in south-eastern Togo. We use several field methods to describe the species richness of mammals in this area, including camera-trapping, recce transects, Kilometric Index of Abundance (KIA) estimates, examination of hunters’ catches and face-to-face hunter interviews. Overall, we directly recorded 19 species that coexist in these forests. Based on interviews, nine other species were confirmed as present in the study area. Only five species were common: *Cephalophus rufilatus*, *Tragelaphus scriptus*, *Chlorocebus aethiops*, *Atilax paludinosus* and *Herpestes ichneumon*. The area still contains various threatened species such as *Tragelaphus spekii* and *Hippopotamus amphibius*. We stress that to ensure the protection of the Dahomey Gap mammals, it is important to seriously consider protecting not only the forest patches but also the surroundings, mainly savannah landscapes.

## 1. Introduction

Habitat fragmentation is one of the main drivers of biodiversity loss in the tropics (e.g., [1,2,3]). In Africa, research on the effects of fragmentation and habitat loss have been limited to tropical forest areas, with much less data on the effects of exploitation and destruction of forest patches on the biodiversity in savannah regions [4,5,6].

The Dahomey Gap is the portion of the Guinean forest-savannah mosaic situated along the West African Gulf of Guinea coast in Benin, Togo, and eastern Ghana. There is evidence that these savannahs were created by deforestation by humans in historical times [7,8]. Large expanses of herbaceous or cultivated land (including urbanized zones) are now interspersed between relatively small forest patches. There are also gallery forests along water courses [7,8].

The Dahomey Gap is exceptional as it separates the forest zone that covers much of the southern region of Benin, Togo, and eastern Ghana into two separate forest blocks, i.e., the Upper Guinean and the Lower Guinean forests [7,8]. Given its unique ecological characteristics, the Dahomey Gap is ideal to study defaunation patterns and changes in animal community structure resulting from forest fragmentation. Data on this issue are relatively scarce, especially studies that document medium and large mammal communities in forest patches in the Dahomey Gap, their relationship with patch size, or how distance from human settlements and other landscape characteristics affect these animal communities.

In this paper we analyse community composition (i.e., species richness), relative abundance (using a kilometric index of abundance), and species–area relationships for medium and large mammals (ungulates, sirenids, primates and carnivores) within a complex of flooded forest patches in the Dahomey Gap in Togo, West Africa. We investigate whether mammal species richness and their relative abundance vary relative to forest patch area and geographic location. For the latter, we considered the distance of a given forest patch to the closest human settlement and to the nearest riverine tract. More explicitly, we answer the following key questions:

(1) What are the most abundant species in the area? We hypothesize that species that are habitat generalists, and thus able to exploit both forest and savannah habitats, should be the most abundant ones.
(2) Does relative forest size and position affect the mammal populations? We hypothesize that, (a) there is a clear forest size–mammal species richness effect; (b) mammal species richness will increase with the distance from the human settlements due to reduced hunting pressure and less habitat disturbance by humans; and (c) closer to rivers, the presence of gallery forests is likely to provide suitable corridors for fauna, which are also less disturbed by humans.

## 2. Materials and Methods

### 2.1. Study Area

The study was carried out in the Avévé forest block (10 km wide in the North, 15 to 20 km in the South) in the Mono Valley, south-eastern Togo (West Africa). This is a riverine basin characterized by small water bodies (ponds) of various sizes but becomes completely flooded in the rainy season. Flooding is more severe when the Nagbéto dam overflows. The climate of the area is subequatorial, with a bimodal regime (two rainy seasons and two dry seasons). Rains are typical from March to July (long rains) and from September to November (short rains), separated by a shorter dry season between July and August and a longer dry season from November to February. The long rainy season concentrates 60% of the precipitation in four months.

**Figure 1:**
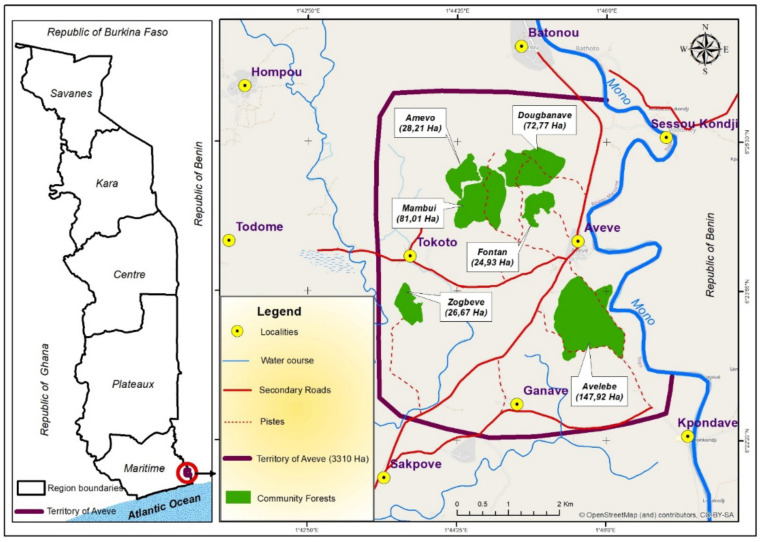
Map of Togo, showing the study area in the south-eastern part of the country.

The area comprises six swamp forest patches of different sizes (Amévo—28.21 ha; Fonta—24.93 ha; Zogbevé—26.67 ha; Mambui—81.01 ha (largely degraded); Dougbanavé—72.77 ha; and Avélébé—147.92 ha) surrounding Avévé village, with three patches located further away from the village: Avélébé to the South, Zogbevé to the West and Amévo to the North (Figure 1). The vegetation in all forest patches is similar, composed of dense thickets of *Morelia africana*, *Drepanocarpus lunatus*, *Qlchornea cordifolia*, *Paullinia pinnata*, *Pterocarpus santalinoides* and *Phoenix reclinata*. Large ponds are found in some patches, especially in Amévo.

All forest patches were surveyed in the study. We also investigated the transition areas, i.e., the spaces between the different forest patches. These open landscapes often contain temporary ponds, fallows and palm groves within a grassland/cultivated landscape that offer suitable habitats for dispersal and are also feeding grounds for medium and large mammals in the region.

### 2.2. Protocol

We employed a multidisciplinary approach to gather information in all forest patches: (i) face-to-face interviews, (ii) field surveys and (iii) camera trapping. Field surveys and interviews were carried out during August–September 2021 by three researchers who accumulated a total of 270 person-hours. Additional time was dedicated to camera-trapping (see below for more details).

We interviewed persons (informally and face-to-face) living in four of the villages—Avévé, Kpondavé, Batonou and Akissa—to generate a list of mammals hunted or known to occur in each of the patches. A total of 15 hunters (aged 25–65 years) were approached, recruited using a snowball procedure. All hunters were informed of the aims of the research study prior to starting the interviews. Every person was interviewed separately, with each interview lasting about one hour. These hunters were also hired to identify signs, tracks or droppings of species in the wild. Interviews allowed us to not just generate a list of species living in the area according to the interviewees, but also identify the hunting practices and the different uses made of hunted prey. We also asked about species that were hunted regularly in the past compared to the present, as well as the number of active hunters, the apparent abundance of the various mammal species, etc.

**Plate 1:**
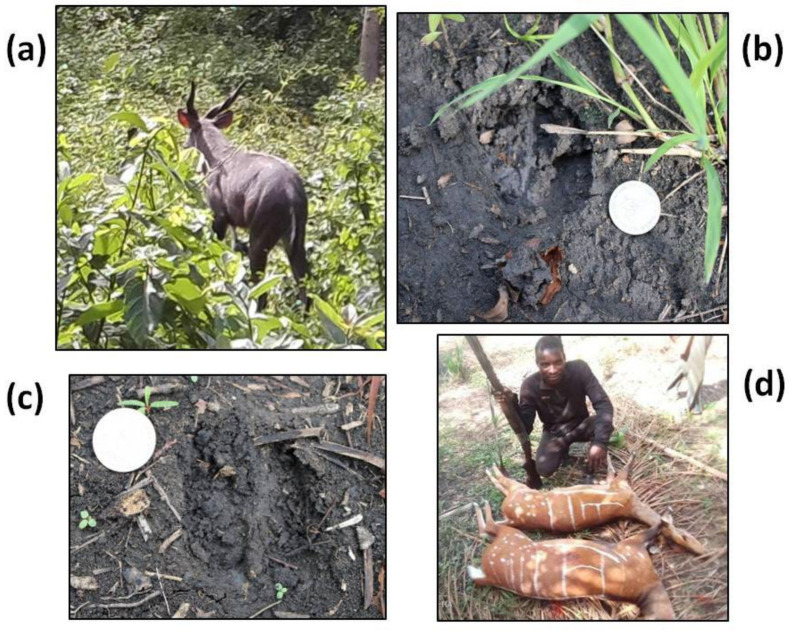
Images of two species of ungulates from the study area: (**a**) *Tragelaphus spekii* male; (**b**) *Tragelaphus spekii* footprint; (**c**) *Tragelaphus scriptus* footprint; (**d**) a hunter with two *Tragelaphus scriptus* individuals.

We considered species present in a certain site only if there was incontrovertible evidence of their existence, e.g., skins, skulls, photographic material or direct observations during our field studies (see below for more details) (Plate 1). By contrast, species reported as present in the past by our interviewees were not included in our analyses.

We carried out field surveys using a combination of techniques. Data on medium and large mammals were collected from (i) camera-trap (photographic trapping), (ii) direct observations of individuals/groups (mainly ungulates and primates) and (iii) recce transects. Camera-trap data were used to demonstrate the presence of a given species but not for calculating its relative abundance. This decision was taken because, after positioning several traps, there was an unexpected high flood from the Nangbeto Dam, and so we were forced to remove all the eventually affected camera traps from their site. So, there were no homogeneous data to be gathered from each trap, preventing any quantitative analyses using them. In the various forest patches, we randomly positioned 100 camera-traps (50 Cuddeback Ambush IR1,187 and 50 Cuddeback Professional Color, model 1347) for a total of 180 days per trap. Overall, 75 traps were placed within the forest habitat and 25 in the surrounding savannah matrix. We installed cameras for monitoring terrestrial species (ungulates, carnivores), positioning them in sites with high concentrations of footprints, scats and other tracks. These sites were identified with the help of local hunters. Each camera was placed 50–90 cm from the ground, and we made sure that the camera’s global view covered the area where the footprints and scats were observed. Camera traps were left unattended in the field for 28 days before being taken to the laboratory and photos examined. To apply the recce method, we formed teams of two people supported by two experienced trackers to reduce noise. Because of logistic constraints, we could not open line transects for this study. Instead, recces were conducted following the paths already created by local hunters. Observation work started very early in the morning (4 h to 11 h) and/or in the late afternoon (15 h to 19 h). The start and end of the censuses were noted to calculate the observation effort as well as the number of kilometres travelled. The team walked at an average speed of 1 km/h searching for signs of mammal activity. When an individual of a species was encountered, the following information was noted: species, time, GPS location, group size and structure, behaviour and habitat type. Other presence indicators were also considered, e.g., smears, footprints (Plate 1), faeces, regurgitations, food remains from feeding spots, etc.

A total of 39.3 km was travelled by each team in the various forest patches. In addition, 3 km were covered along the Mono river by canoe to record feeding signs of the West African manatee *Trichechus senegalensis*. The observation effort for the entire mission in each forest patch was 37 person-hours.

### 2.3. Statistical Analyses

We used the Kilometric Index of Abundance (KIA) to describe the relative abundance of the various species in the study area. A UPGMA dendrogram, with a Euclidean similarity index and 40 bootstraps to compute branch measurements, was completed to evaluate the dissimilarity among the various forests in terms of the presence/absence of the mammal species recorded in the study area.

To assess the effects of (i) area (in ha) of the forest patch (AREA), (ii) its distance (km) from the Mono river (DISTRIV) and (iii) its distance (km) from the nearest human settlement (DISTVIL) on the species richness at each site, we used polynomial equation models (linear, order 2, order 3, order 4), and then selected the best model based on the Akaike Information Criterion for small samples (AICc), with the model showing the lowest AICc value being the selected model. AREA, DISTRIV and DISTVIL were calculated with GIS, using the barycentre of the forest patch as the origin point for the measurements. The correlation between forest patch area (ha) and mean body size (mass, in g) of the mammal species inhabiting each patch was assessed by a Spearman’s rank correlation coefficient. All analyses were performed with PAST 4.0 statistical software, with alpha set at 5%.

## 3. Results

### 3.1. General Data

The number of dedicated active hunters working in the forest patch system was 10, with additional individuals hunting in the forests less regularly. We obtained presence data for 19 species of medium and large mammals (Table 1). Six species were directly observed in the field, six as carcasses in the villages, five by camera-trapping, five by footprints, three from feeding signs and five from scats (Table 1). Fourteen species were also recorded by face-to-face interviews, including eight species that were also observed with at least one other method. Excluding the species that were recorded only by interviews (n = 6), one species was recorded only by village carcasses, one only by scats and one only by footprints, while most of the species were recorded by multiple methods (Table 1).

The number of species found in each forest patch ranged between 8 and 19, with Avélébé being by far the area with the highest species diversity (Table 1). Eight species were recorded in all forest patches, whereas five species (*Mellivora capensis*, *Leptailurus serval*, *Atilax paludinosus*, *Philantomba walteri* and *Hippopotamus amphibius*) were only found in a single forest patch, Avélébé (Table 1).

**Figure 2:**
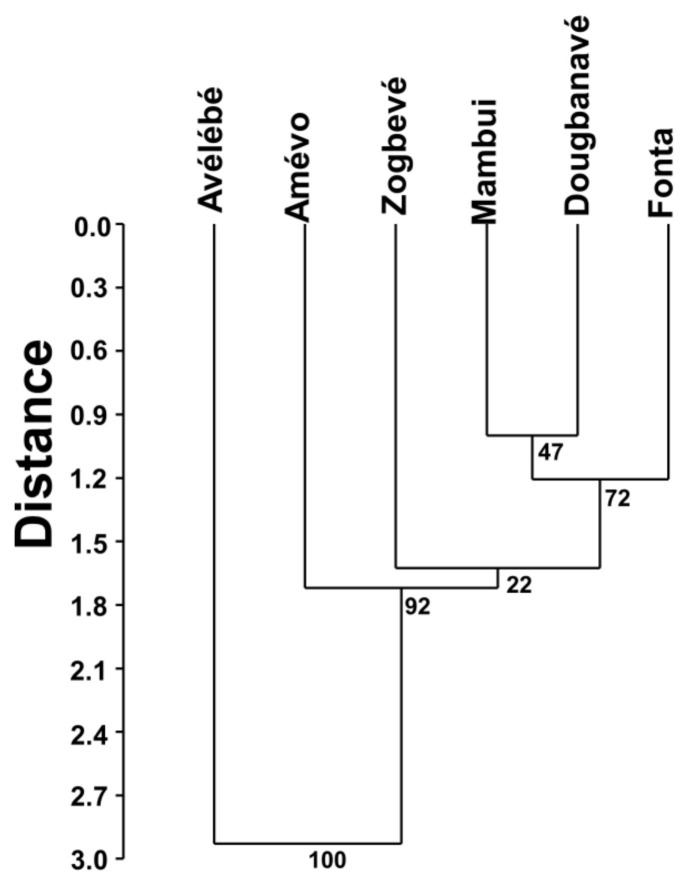
UPGMA dendrogram, with a Euclidean similarity index and 40 bootstraps to compute branching measurements, of the dissimilarity among the various forests in terms of the presence/absence of the mammal species recorded at the study area in south-eastern Togo.

The UPGMA dendrogram revealed that Mambui, Dougbanavé and Fonta were relatively similar in terms of their medium and large mammal fauna, whereas the other three forests were very different from these three and from each another (Figure 2).

**Figure 3:**
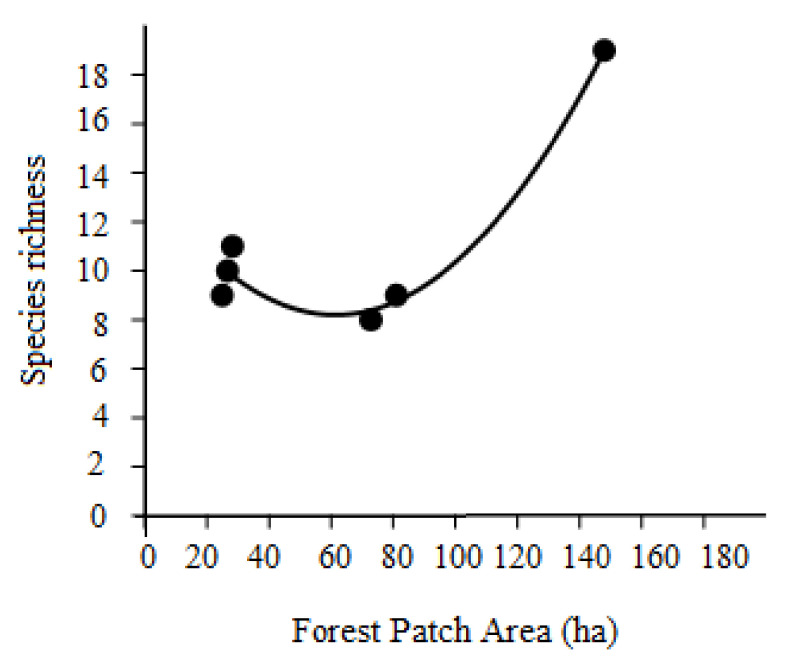
Order-2 polynomial relationship between forest patch area and species richness of medium and large mammals in the study area.

Despite the very small number of cases (*n* = 6), there was a statistically significant positive effect of AREA on species richness (order-2 polynomial model: number of species = 0.001327 × AREA^2^ + 0.165 × AREA + 13.4; F = 32.86, P = 0.00912, AICc = 20.917) (Figure 3). On the contrary, the effect of DISTVIL on species richness was positive but non-significant (linear model, F = 0.617, P = 0.476, AICc = 65.9) and that of DISTRIV was negative and non-significant (order-2 polynomial model, F = 2.189, P = 0.259, AICc = 45.2). There was no effect of the patch area on the average body size of the mammal species inhabiting it (Spearman’s rank correlation rs = 0.029, P = 0.960) and the mean body sizes were similar across patches (slightly larger in Avélébé, although data not reported for brevity).

Table 2 presents the KIA estimates for the 13 mammal species observed directly during the present study. Species reported as present by interviewees, seen in camera trap photo or as carcasses in the villages (see below) were not seen in our field recces, and therefore their KIA abundance could not be calculated. Surprisingly, the highest KIA was calculated for *Trichechus senegalensis*, a threatened species. However, this estimate cannot be compared with those of land species. Only four terrestrial species had relatively high KIA estimates: *Chlorocebus tantalus* and *Herpestes ichneumon* were the most common species, followed by *Tragelaphus scriptus* and *Erythrocebus patas* (Table 2).

### 3.2. Species Directly Recorded

The summarized information on the 13 mammal species that were directly recorded by sightings and/or recce methodology is shown in Online Supplementary Table S1. Overall, considering the frequency and spatial distribution of our direct observations, the number of specimens found in villages and the information obtained from hunters, only five species of medium and large mammals can still be considered common in the study sites: *Cephalophus rufilatus*, *Tragelaphus scriptus*, *Chlorocebus aethiops*, *Atilax paludinosus* (although at a single forest patch) and *Herpestes ichneumon*. Numerous trophies and carcasses of *T. scriptus* were seen in hunter settlements bordering both the Avévé and Akissa forests (Plate 1), indicating that this species is or was widespread in south-eastern Togo.

### 3.3. Species Confirmed by Evidence during Interviews

Six additional species were confirmed as being present in the study area from interviews: (i) *Hippopotamus amphibius*, currently rare and confined to a single patch within the study area, with various individuals killed between Avévé and Agbanakin about 10 years ago; (ii) *Philantomba walteri*; (iii) *Perodicticus potto juju*, whose presence in three forest patches is certain as these species are hunted regularly or exported for the international pet trade by operators of wild animal farms in Lomé; (iv) *Galagoides demidovii*, also described in all the six forest patches and was exported between 1980 and 2010 (*n* = 934 specimens) from Togo; this species was most often collected from the Hadjivi, Avévé and Akissa forests; (v) *Civettictis civetta*; and (vi) *Mellivora capensis*. This latter species is very rare in the Avévé forests according to hunters, but its calls were unambiguously heard at night in the Akissa forest [9].

Apart from the above-mentioned species, hunters suggested that the duiker *Sylvicarpa grimmia* as well as *Kobus kob* has been seen, which are species that had apparently been extirpated in the Avévé forest. According to various hunters interviewed, their local extinction was because during flood periods these species sought refuge in areas close to human settlements, where they were easily hunted. The last individuals of these species were hunted during floods between 2008 and 2010. Long before the 2000s, populations of buffaloes (*Syncerus caffer*) and bongo (*Tragelaphus erycerus*) disappeared from the Avévé forests. The populations of these two species are now confined to the Togodo protected areas complex. During the 2000s, a solitary bongo from Togodo was killed by hunters in the Avévé forest.

## 4. Discussion

### 4.1. Is the Species Richness of Each Forest Patch Low Compared to the Overall Species Richness of the Whole Region?

Our surveys identified a total of 19 medium and large mammal species, showing a relatively poor species diversity compared to the much higher number of species known to occur in this part of Togo and in Dahomey Gap habitats [10]. None of the detected species is new for Togo [10]. However, in our analyses we did not consider rodents and bats, which by far account for the largest diversity of mammal species in the country [10]. Ecologically, the observed assemblage of mammals is characteristic of wetland ecosystems in the Dahomey Gap. Indeed, given the massive habitat alteration that has occurred throughout the whole region, Togo’s coastal wetland forest system along the Mono river remains the area where a few individuals of several endangered species continue to survive. For instance, hippopotamuses are today only found in the Afito pond, whereas this species was widely distributed throughout southern Togo and even in Lake Togo, Haho and Zio until a few decades ago [10].

Our study also pointed out that to investigate the mammal fauna of the forest patches of the Dahomey Gap, it is necessary to apply a suite of different methods. In fact, while most of the species (the least elusive ones) can be easily detected with several methods, there is not any single method that allowed the detection of all the species found in the area. The use of Local Ecological Knowledge through face-to-face interviews was also instrumental to accurately describe the mammalian species richness of the area, given that six species were recorded only using this method and 73.7% of the species that were directly observed in the field (by camera traps, scats, footprints, etc.) were also described as present by our interviewees.

### 4.2. Is There Any Effect of Relative Size and Position of the Various Forest Patches on the Mammal Populations?

The observed affinities between the six flooded forest patches, as highlighted by the UPGMA analysis, showed that the relative position of the various forests with respect to the Avévé village is crucial to define their faunal characteristics, whereas our polynomial models revealed that the area of the forest patch had a higher influence on the species richness than distance from the nearest human settlement or river. Species–area relationships are among the strongest empirical generalizations in community ecology theory [11,12,13,14], thus the area effects we observed on mammal species richness in the Dahomey Gap are not surprising and have already been recorded previously for mammals in different habitats (e.g., see [15,16]), including large mammals in African savannahs [17] and forests [18,19].

The effect of the relative location of the various forest patches is clear since the largest and most isolated of these (Avélébé) still contained by far the highest diversity of medium and large mammals. Moreover, the other two forest patches situated farther away from Avévé village (i.e., Amévo and Zogbevé) differed in terms of mammal species presences/absences compared to the three forests closest to the Avévé village (Fonta, Mambui and Dougbanavé). These were instead very similar to each other. As for the Avélébé forest, its higher species richness compared to the other fragments may partially be due to its proximity to the Mono river, which may allow mammal populations to disperse more easily and move into other forested patches or gallery forests along the banks of this river. Overall, the pattern of faunal similarity between the various forest areas, which correlated with area and their respective locations relative to the Avévé village, supports the notion that: (1) there is strong hunting pressure from the population of the village itself and (2) that such hunting activity clearly shaped the structure of the medium and large mammal community of the study area. In this regard, humans can therefore significantly influence the species–area relationships for mammals in African tropical ecosystems [18,19]. It is also interesting to note that the various forest patches, although very close to each other (on average about 2 km distance) differed in their mammal communities. Since the linear distances between patches are far too small to prevent movements among them for foraging reasons throughout the year (i.e., the search of different sources of seasonal food), we think that it is the nature of the matrix (very disturbed in most sites) that represented a barrier to the dispersal of these animals. These observed patterns are consistent with the general notion that extinction risks in terrestrial mammals are correlated with habitat fragmentation [20], and therefore with patch size and the relative extent of the matrix between the nearby forest patches [21,22,23]. However, patch size is not always associated with mammal species persistence in wooded and forested habitats, as demonstrated for the Eastern Bettong (*Bettongia gaimardi*) in fragmented habitats [24].

Different detection probabilities may have partially biased our presence data and KIA estimates in the various habitats [25,26,27]. Thus, our data should be considered as preliminary and need further research for a better understanding of the patterns involved. 

Indirect evidence of the hunting pressure on mammals is the fact that the majority of the individuals and their tracks were mostly found in highly vegetated localities. For instance, footprints and droppings of Sitatunga (*Tragelaphus spekii*), one of the rarest species, were found in a site with dense *Drepanocarpus lunatus, Qlchornea cordifolia* and *Pterocarpus santalinoides* (Plate 1). Previous studies showed that mammals tend to modify their habitat use to minimize hunting risks in fragmented landscapes [28], thus our data fit with this general evidence.

Another piece of indirect evidence of the heavy hunting activity is that many hunters were able to consistently describe the seasonal movements of the various species between forest patches, thus showing good knowledge of their habits. For instance, various hunters independently reported that (i) Sitatunga individuals often disperse into the various forest patches of Avévé including Akissa and Tetekondji; (ii) *Potamochoerus porcus* individuals make seasonal trips between the villages of Agome and Glouzou, at the mouth of the Mono in Benin; (iii) *Cercopithecus mona* groups regularly travel between the forests of Zogbevé, Avélébé and Akissa; and (iv) *Leptailurus serval* can be found in Avévé, Akissa and Tetekondji, with possible migration of individuals from Togodo to Avévé. Although our data does not allow us to verify the accuracy of this information, the fact that it has been reported by several hunters independently suggests that it is reliable. If this is the case, the role of the network of forest patches in maintaining the richness of mammalian species in the study area is evident, given that they would use not simply a single patch but the set of forested patches according to their ecological needs. It is therefore also important to monitor and manage the environmental matrix between the various forest patches, given that it is particularly exposed to human impact from agriculture, settlements and roads, but are used by medium and large mammals which move from one forest patch to another.

### 4.3. What Are the Most Abundant Species in the Area?

Our surveys suggest that individuals of only five terrestrial species were frequently encountered, and consequently had high relative KIA abundance estimates: *Cephalophus rufilatus*, *Tragelaphus scriptus*, *Chlorocebus aethiops*, *Atilax paludinosus* and *Herpestes ichneumon*. All these species are characterized by a wide distribution and the ability to inhabit both savannah and open forest vegetation zones, with *A. paludinosus* being a strictly riverine species. Conversely, specialist forest species were either very rare (as in the case of the Sitatunga) or even locally extinct (e.g., bongo). Thus, our prediction that generalist species should be favoured in the Dahomey Gap forest patches was empirically confirmed by the data.

### 4.4. Conservation Considerations

From our observations we suggest that to preserve mammal populations of the Dahomey Gap, it is not enough to protect the integrity of the forested patches, but it is also essential to monitor and protect the large, grassy, savannah-like vegetation between these patches, which are used by animals to move between forest patches during certain seasons. We suggest that any management plan currently being developed must consider the full protection of the Avévé forest as well as other associated ecological units (such as the sacred forest of Akissa). The Fonta, Amévo and Avélébé forests should be considered core sites in any ecologically based development plan for the study area. More generally, the continuum network of sacred and community forests, and of their grassy boundaries, should receive priority attention by the competent authorities [29,30].

## 5. Conclusions

It is crucial to protect the forest–savannah mosaics in order to ensure that the ecological dynamics (inter-patch routes, seasonal movements) of the Dahomey Gap mammals are maintained. This protection should include the buffer zones and not just the forest patches, given that the savannah-like buffers are also important elements of the habitat niche of a variety of species during at least some periods of the year [31].

## Figures and Tables

**Table 1:** Presence of the various species of mammals in the various forest blocks at the study area and details of the method by which each species was recorded.

	Method	Fonta	Mambui	Dougbanavé	Zogbevé	Amévo	Avélébé
*Chlorocebus aethiops tantalus*	DO, CT	x	x	x	x	x	x
*Cercopithecus mona*	DO, FS					x	x
*Erythrocebus patas*	FC, DO				x	x	x
*Galagoides demidovii*	IN	x	x	x	x	x	x
*Galago senegalensis*	VR, IN	x	x	x	x	x	x
*Perodicticus potto juju*	IN		x			x	x
*Genetta tigrina*	FC, IN	x	x	x	x	x	x
*Herpestes ichneumon*	CT, DO, FC	x	x	x	x	x	x
*Civettictis civetta*	IN	x	x	x	x	x	x
*Mellivora capensis*	IN						x
*Leptailurus serval*	FP, IN						x
*Atilax paludinosus*	FP, DO, VR, FC						x
*Potamochoerus porcus*	FS, VR, IN				x		x
*Tragelaphus scriptus*	FP, CT, DO, VR, FC, IN	x	x	x	x	x	x
*Tragelaphus spekii*	FP, CT, IN	x					x
*Cephalophus rufilatus*	FP, VR, IN	x	x	x	x	x	x
*Philantomba walteri*	IN						x
*Hippopotamus amphibius*	IN						x
*Trichechus senegalensis*	FS, VR, IN						x
TOTAL		9	9	8	10	11	19

Legend: FS = feeding signs; CT = camera trapping; DO = direct observation; FP = footprints; FC = faeces; VR = village records; IN = face-to-face interviews.

**Table 2:** Diversity and Kilometric Index of Abundance (KIA) estimates for the various species of mammals in the Avévé forest block.

	No. Individuals	Travelled Distance	KIA
*Atilax paludinosus*	10	39.3	0.25
*Cephalophus rufilatus*	5	39.3	0.127
*Cercopithecus mona*	2	39.3	0.05
*Chlorocebus aethiops tantalus*	36	39.3	0.916
*Erythrocebus patas*	15	39.3	0.382
*Genetta tigrina*	1	39.3	0.025
*Herpestes ichneumon*	25	39.3	0.636
*Leptailurus serval*	1	39.3	0.025
*Potamochoerus porcus*	2	39.3	0.05
*Tragelaphus scriptus*	17	39.3	0.433
*Tragelaphus spekii*	2	39.3	0.05
*Trichechus senegalensis*	2	3	0.666

## Data Availability

All data collected are available in the present paper.

## Supplementary Materials

The following supporting information can be downloaded at: https://www.mdpi.com/article/10.3390/ani12213041/s1, Table S1: Synopsis of the direct sightings of the medium and large mammal species that were observed at the study area and relevant notes. For common species that were observed in multiple places, only sites of special ecological relevance (for instance, where various individuals were seen) are provided in this table.
