# Supplementary material for: Diversity and Relative Abundance of Ungulates and Other Medium and Large Mammals in Flooded Forests in the Dahomey Gap (Togo)
Source: Animals (Basel). 2022 Nov 4;12(21):3041. doi: 10.3390/ani12213041 (PMC9654704; doi:10.3390/ani12213041)
Supplement: Supplementary file 1 [file animals-12-03041-s001.zip › animals-1999909-supplementary.pdf]

SUPPLEMENTARY MATERIALS

Table S1. Synopsis of the direct sightings of the medium and large mammal species that were observed at the study area and relevant notes. For common species that were observed in multiple places, only sites of special ecological relevance (for instance, where various individuals were seen) are provided in this table.

species	longitude	Latitude	notes
Tragelaphus spekii	E 01°45′16.6″	N 06°24′49.0″	site with dense *Drepanocarpus lunatus, Qlchornea cordifolia* and *Pterocarpus santalinoides*
Tragelaphus scriptus	E 01°44′47.4″	N 06°24′35.8″	Most of the populations occur in just three sites: the forest of Fonta, the forest of Amévo and the forest of Avélébé, all being characterized by temporary ponds with *Drepanocarpus lunatus*
Cephalophus rufilatus	E 01°45′16.6″	N 06°24′49.0″	footprints; regularly hunted in all the villages bordering Avévé
	E 01°44′30.2″	N 06°24′40.8″	footprints; regularly hunted in all the villages bordering Avévé
	E 01°45′00″	N 06°25′11.2″	footprints; regularly hunted in all the villages bordering Avévé
Potamochoerus porcus			Avélébé; hunted individuals seen
Trichechus senegalensis	E 1°46′32.06″	N 6°24′21.15″	reported by hunters to make seasonal trips between the villages of Agome and Glouzou
	E 1°46′05.13″	N 6°23′53.97″	reported by hunters to make seasonal trips between the villages of Agome and Glouzou
Cercopithecus mona	E 01°44′01.2″	N 06°23′36.4″	reported by hunters to regularly travels between Zogbevé, Avélébé and Akissa forests
Chlorocebus aethiops			observed several times during our surveys in all forest patches; common in the Avévé forest
Galago senegalensis	E 01°44′33.6″	N 06°24′38.5″	
Leptailurus serval	E 01°45′22.0″	N 06°23′01.5″	footprints; according to hunters it can be found in the forests of Avévé, Akissa and Tetekondji
Genetta tigrina	E 01°45′11.5′	N 06°24′38.7″	regularly hunted in all forest patches of Avévé
Atilax paludinosus	E 01°44′01.9″	N 06°23′37.3″	footprints and droppings were observed in several spots at Avélébé
Herpestes ichneumon	E 01°45′02.8″	N 06°24′27.9″	regularly hunted in all forest units of Avévé, a sit is feared by the villagers for its attitude to enter houses to hunt chickens if prey becomes scarce
	E 01°44′51.6″	N 06°25′00.3″	regularly hunted in all forest units of Avévé, a sit is feared by the villagers for its attitude to enter houses to hunt chickens if prey becomes scarce
